# Effect of spatial configuration of an extended nonlinear Kierstead–Slobodkin reaction-transport model with adaptive numerical scheme

**DOI:** 10.1186/s40064-016-1941-y

**Published:** 2016-03-09

**Authors:** Kolade M. Owolabi, Kailash C. Patidar

**Affiliations:** Department of Mathematics and Applied Mathematics, University of the Western Cape, Bellville, Republic of South Africa

**Keywords:** Exponential time differencing Runge–Kutta method, Diffusion-driven, KiSS model, Spatial pattern formation, Stability, 92D25, 92D40, 65L06, 65M12, 65M20

## Abstract

In this paper, we consider the numerical simulations of an extended nonlinear form of Kierstead–Slobodkin reaction-transport system in one and two dimensions. We employ the popular fourth-order exponential time differencing Runge–Kutta (ETDRK4) schemes proposed by Cox and Matthew (J Comput Phys 176:430–455, 2002), that was modified by Kassam and Trefethen (SIAM J Sci Comput 26:1214–1233, 2005), for the time integration of spatially discretized partial differential equations. We demonstrate the supremacy of ETDRK4 over the existing exponential time differencing integrators that are of standard approaches and provide timings and error comparison. Numerical results obtained in this paper have granted further insight to the question ‘What is the minimal size of the spatial domain so that the population persists?’ posed by Kierstead and Slobodkin (J Mar Res 12:141–147, 1953), with a conclusive remark that the population size increases with the size of the domain. In attempt to examine the biological wave phenomena of the solutions, we present the numerical results in both one- and two-dimensional space, which have interesting ecological implications. Initial data and parameter values were chosen to mimic some existing patterns.

## Background

The study of nonlinear reaction–diffusion equation of the form

1 $$\frac{\partial u}{\partial t} = D \nabla^{2} u + f(u) \quad {\rm for} \; 0 < x < l, \quad t > 0$$

2 $$u(x,0) \, = d(x)$$

3 $$u(0, \, t) \, = a(t)$$

4 $$u(l, \, t) \, = b(t)$$

has a long-standing history in mathematical modeling of propagation phenomena that mostly occurs in distributed dissipative dynamics, as well as diffusive transport of mass and heat, there is some reaction term representing, for instance, population growth or heat generation. Most realistic physical problems such as Allen-Chan, Burgers, Cahn–Hilliard, Fisher-KPP, Nagumo, Gray-Scott (or cubic autocatalytic), Kierstead, Slobodkin and Skellam (KiSS), Kuramoto–Sivashinsky and host of others, naturally exist in form of higher-order partial differential equations. In Holmes et al. (1994), PDEs of the class (1) have shown to provide a natural framework for investigating the influence patch size and geometry on the population dynamics of organisms living within an habitat. Many researchers have used equations of the form (1) in different forms especially in relation to three applications that model the behavior of biological systems in a spatial setting. The three major and popular applications of reaction–diffusion models relate to critical patch size (Kierstead and Slobodkin 1953), spread of advantageous genes (Fisher 1937), and pattern formation (Turing 1952). For instance, if the reaction or interaction term *f*(*u*) is replaced by *κu*(1 − *u*), where *κ* and *D* are positive parameters regarded as the carrying capacity and diffusion respectively in the context of biology, then (1) becomes the classic simplest case of a nonlinear reaction–diffusion equation popularly referred to as Fisher equation (1937) with history dated back to 1937, which has since becomes one of the most well-studied reaction–diffusion models in population biology to describe the spread of an advantageous allele.

In addition, A large number of nonlinear reaction–diffusion PDEs of the form (1), sometimes exist in the form of blow-up problems if $f(u) = u^{\alpha } ,\,\,\,\alpha > 0$. In PDEs systems, solution with the given initial data often lead to singularity (which could either be a point where a discontinuity occurs or the dependent variables tend to infinity) in finite time, such a phenomenon is widely referred to as the blow-up, and the time at which such occurs is known as the blow-up time, see for instance, (Owolabi 2015b; Roberts 2007) and references therein. A generalization of (1), which frequently occurs as a limiting case of a system, is when there is nonlocal dependency, often of the source term, upon *u*, we do not treat this problem here. We briefly present a review in “An extended KiSS model” section for special nonlinear KiSS model.

It is important to check for the uniqueness of solution of the class of diffusion Eq. (1).

### **Proposition 1**

*The Dirichlet problem* (1)–(4) *has at most one solution.*

### Proof

Let us adopt energy integral method for this proof, by assuming that there exist two solutions *u*_*α*_ (*x*, *t*) and *u*_*β*_ (*x*, *t*); let *u* = *u*_*α*_ − *u*_*β*_. Based on the definition, *u* satisfies

$$u_{t} - Du_{xx} = 0, \quad {\rm for} \; 0 < x < l\quad {\text{and}} \quad t > 0,$$

$$u\left( {x,0} \right) = 0,\quad u(0,t) = 0 \quad {\text{and}} \quad u(l,t) = 0.$$

Furthermore, we obtain

$$0 = (u_{t} - Du_{xx} )u = \left( {\frac{{u^{2} }}{2}} \right)_{t} + ( - Du_{x} u)_{x} + Du_{x}^{2} .$$

On integration, we get

$$0 = \int\limits_{0}^{l} {\left( {\frac{{u^{2} }}{2}} \right)}_{t} dx - \left[ {Du_{x} u} \right]_{0}^{l} + D\int\limits_{0}^{l} {u_{x}^{2} dx} .$$

The second term to the right is zero from the boundary condition, thus we can take the *t* derivative as

$$\frac{d}{dt} = \int\limits_{0}^{l} {\frac{{\left( {u(x,t)} \right)^{2} }}{2}dx = - D\int\limits_{0}^{l} {\left( {u_{x} (x,t)} \right)^{2} dx \le 0} } .$$

This implies that the integral $\int_{0}^{l} {}$ is monotonically decreasing in *t*, meaning that in each case we expect to have

$$0\le \int\limits_{0}^{l} {\left( {u(x,t)} \right)^{2} } dx \le \int\limits_{0}^{l} {\left( {u(x,0)} \right)^{2} } dx = 0.$$

So, it follows that *u*(*x*, *t*) = 0, *u*_*α*_ = *u*_*β*_ for all *t* ≥ 0.□

We can also obtain the qualitative information based on the time of local existence of solution. By considering the system (1) in a single space variable *u* = (*u*_1_, *u*_2_,*…*, *u*_*n*_), *D* = diag(*d*_1_, *d*_2_, *…*, *d*_*n*_), with *d*_*i*_ ≥ 0 for all *i*. We also in addition consider the initial data

5 $$u\left( {x,0} \right)=u_{0} \left( x \right), \quad x \in Rn.$$

Next, solutions that are continuous function of time can be obtained in Banach spaces. We proceed by using definition to describe these space. Let *B* denote a Banach space of functions in **R**^n^ → **R,** let ∥·∥_∞_ denotes *L*_*∞*_-norm, and ∥·∥_*B*_.

### **Definition 2**

B is acceptable if the following conditions hold:

(i) B is a bounded subset and continuous functions on *R*, and if *v* ∈ *R*, then ∥*v*∥_*B*_ ≥ ∥*v*∥_∞_.
(ii) *B* is the translation-invariant. That is, *voϱ* ∈ *B* for every *v* ∈ *B* and every translate *ϱ*:*R* → *R*. Also ∥*voϱ*∥_*B*_ = ∥v∥_*B*_.
(iii) If *R*^*n*^ → *R*^*n*^ is smooth, and *f* (0) = 0, then *f* (*v*) ∈ *B* for every *v* ∈ *B*, and for any *G* > 0, there exists a constant *k* (*G*) such that
  $$\| f\left( G \right) - f\left( {G^{{\prime }} } \right)\|_{B} \le k\left( G \right)\| v{-}v^{{\prime }} \|$$
  for all *v*, *v*′ in *B* with ∥*v*∥_∞_, ∥*v*′∥_∞_ ≤ G.
(iv) If *ϱh*:*R* → R represents translation by *h*, that is *ϱh* (*x*) = *x* + *h*, then for each *v* ∈ *B*, we have
  $$\| vo{\varrho} h - v\|_{B}\, \to\, 0\quad {\text{as}}\;h \to 0.$$

The aims of this work are in folds. We first give an extension to the existing linear KiSS model in the form of a nonlinear type. Secondly, we formulate a viable numerical techniques in space and time for the numerical simulation of an extended KiSS model in one and two dimensional spaces. We finally justify the suitability and applicability of the present numerical techniques with the existing family of higher-order time-stepping schemes.

The rest of paper is structured as follows. In “An extended Kiss model” section, an extension is given to the KiSS model with underlying theory to back the patchy size selection. We discuss adaptive numerical methods in space and time as well as the stability analysis of the scheme in “Numerical method” section. Numerical experiments in one- and two dimensions are examined in “Numerical simulations” section. Finally, conclusion is drawn in “Conclusion” section.

## An extended Kiss model

In the present paper, numerical solution of an exponential growth model of the form (1), where the reaction term *f* (*u*) is given as *τ uα*, so that Eq. (1) becomes

6 $$\frac{\partial u}{\partial t} = D{\nabla }^{2} u + \tau u^{\alpha }$$

where the diffusion coefficient *D*, the growth rate *τ* and the critical exponent constant *α*, are all positive parameters. This equation is the critical patch model popularly known as the KiSS model named after Skellam (1951) and Kierstead and Slobodkin (1953) which was originally developed to describe the spread of red tide outbreaks. Red tide is a name given to the discolored waters caused by the aggregation or blooming of microscopic organisms. A model for growth and spread of a population is used to determine the minimal size of the spatial domain needed for population to survive and this minimal size is is referred to as the critical patch size (Allen 2007).

In the classical paper (Kierstead and Slobodkin 1953), the critical patch size was determined for a simple reaction–diffusion equation with exponential growth, their model was applied to study phytoplankton plants living in the ocean. Determination of patch size of one-dimensional form of (6) have been considered (Allen 2007; Kierstead and Slobodkin 1953; Kot 2001; Murray 1989; Okubo 1978) on the spatial domain [0, *l*] via separation of variables method. In one-dimension with the choice *α* = 1, we have the KiSS model

7 $$\frac{\partial u}{\partial t} = D\nabla^{2} u + \tau u,\quad 0 \le x \le l,\quad t > 0 ,$$

Subject to initial and homogeneous boundary conditions

8 $$u(x,0) = u_{0} (x),\quad 0 \le x \le l ,$$

9 $$u(0,t) = u(l,t) = 0,\quad t > 0 ,$$

where *D* > 0 and *τ* > 0. The solution is given as

10 $$u(x,t) = \sum\limits_{n = 1}^{\infty } {a_{n} \sin \left( {\frac{n\pi x}{l}} \right)} \exp \left[ {\tau - \frac{{n^{2} \pi^{2} }}{{l^{2} }}} \right]t$$

with

11 $$a_{n} = \frac{2}{l}\int_{0}^{L} {u_{0} (x)\sin \left( {\frac{n\pi x}{l}} \right)} dx$$

By examining the solution reveals the condition that supports population growth and extinction.

For instance, if

$$l < \pi \sqrt {\frac{D}{\tau }} ,$$

then *u*(*x*, *t*) will approach zero as time progresses, while if

$$l > \pi \sqrt {\frac{D}{\tau }} ,$$

*u*(*x*, *t*) will increase indefinitely with time, thus leading to the bloom of the plankton.

An attempt to have a better understanding of how the solution of the reaction–diffusion Eq. (6) behaves, we let *τ* = 0. Hence, Eq. (6) reduces to diffusion equation. We can now find the solution of the general initial value problem of solving (6) in spatial variable *x*, subject to

12 $$u(x,0) \, = u_{0} (x), \quad {\rm for} - l \le x \le l,$$

with the aid of the Fourier transforms, as then *u*(*x*, *t*) will approach zero as time progresses, while if

13 $$u(x,t) = \frac{1}{{\sqrt {4\pi Dt} }}\int_{ - l}^{l} {u_{0} (X)} \exp \left\{ {\frac{{\left( {x - X} \right)^{2} }}{4Dt}} \right\}dX .$$

On using the initial condition as the localized source of the spread of species population, *u*0(*x*) = *δ*(*x*), then, (13) becomes

14 $$u(x,t) = \frac{{e^{{ - x^{2} /4Dt}} }}{{\sqrt {4\pi Dt} }} .$$

As time increases the solution spreads out, having a typical width of $O\left( {\sqrt {4\pi Dt} } \right)$ and a maximum height of $1/\sqrt {4\pi Dt}$. It is also noticeable that the diffusion transports the species within the interval of integration [−*l*, *l*], since *u*(*x*, *t*) > 0 for all *x* when *t* > 0. For *|x|* ≫ 1 and *t* ≪ 1, the corresponding species concentration are very small. If *u*_0_(*x*) = *G*(−*x*), then the solution takes the form

15 $$u(x,t) = \frac{1}{\sqrt \pi }\int_{{x/\sqrt {4Dt} }}^{l} {e^{{ - \xi^{2} }} } d\xi .$$

Further, we consider the reaction–diffusion system (7) on (*x*, *t*) *∈* Ω × R+ , for Ω is defined as a bounded domain in R^*m*^, *∂*Ω is smooth, *u ∈* R^*n*^, and *f* (*u*) = *τ* (*u*) is smooth in *U* *⊂* R^*n*^ *→* R^*n*^ for each *t* ≥ 0. D is nonnegative diagonal matrix of size *n* × *n*. So, we assume that system (7) has a bounded invariant region

16 $$\sum { = \prod\limits_{1}^{n} {\left[ {a_{i} ,b_{i} } \right]} } ,$$

where −*∞* < *a*_*i*_ < *b*_*i*_ < *∞*, *i* = 1, 2,*…*, *n*. In addition to (7), we have the initial conditions

17 $$u(x,0) = u_{0} (x) = \left( {u_{1}^{0} ,u_{2}^{0} , \ldots ,u_{n}^{0} } \right)(x)$$

where *u*_0_(*x*) lies in Σ*∀x* *∈* Ω. We also assume that *u* is admissible of the homogeneous Neumann boundary conditions

18 $$\frac{du}{dt} = 0\quad {\text{on}} \; \partial \Omega \times {\rm R}{+} .$$

### **Lemma 3**

*Let* Σ *be defined by* (16) *and ρ* = *ρ*(*u*, *t*) *is Lipschitz continuous in* Σ, *for each t* ≥ 0*. If*

$$\rho^{ + } (u, \, t) \, = \, \sup \{ \rho (u_{1} , \, \xi_{2} , \, \xi_{3} , \ldots ,\xi n,t):a_{i} \le \xi_{i} \le u_{i} , \, i = \, 2,3, \ldots ,n\}$$

*Then ρ*^+^*is Lipschitz continuous in* Σ.

### *Proof*

Let us assume that *a*_*i*_ = 0, *i* = 1, 2,*…*, *n*. For any *u ∈* Σ, the set

$$L_{u} = \{ \xi \in {\mathbf{R}}^{n} : \, 0 \le \xi_{j} \le u_{j} , \, j \, > 1, \, u_{1} = \xi_{1} \}$$

then *ρ*^+^(*u*, *t*) = sup{*ρ*($\xi$, *t*): $\xi$* ∈ Lu*}. If we let *u*, *z ∈* Σ, and since *ρ* is a continuous, there exists $\xi^\prime$*∈ L*_*u*_ so that

$$\rho^{ + } (u, \, t) \, = \rho ({\xi^\prime} , \, t).$$

If *ξ*_0_ = max(0,$\xi^\prime$ + *z* − *u*), that is, ($\xi^\prime_0$)_*j*_ = max(0, $\xi^\prime_j$ + *z*_*j*_ − *u*_*j*_), *j* = 1, 2,*…*, *n*. It is obvious that $\xi^\prime_0$ *∈* *L*_*z*_, and that

19 $$|\xi^{{\prime }}_{0} - \xi^{{\prime}} \left| { \, \le \, } \right| u - z|.$$

Known that

$$|\xi_{0}^{{\prime }} - \xi^{{\prime }} |^{2} = \Sigma [\xi^{{\prime }}_{j} - \hbox{max} (0, \, \xi^{{\prime }}_{j} + z_{j} - u_{j} )]^{2} ,$$

and if $\xi^\prime_j$ + *z*_*j*_ − *u*_*j*_ ≥ 0, $\xi^\prime_j$ − max(0, $\xi^\prime_j$ + *z*_*j*_ − *u*_*j*_) = *u*_*j*_ − *z*_*j*_, while $\xi^\prime_j$ + *z*_*j*_ − *u*_*j*_ < 0, then $\xi^\prime_j$ − max(0, $\xi^\prime_j$ + *z*_*j*_ − *u*_*j*_) = $\xi^\prime_j$ < *u*_*j*_ − *z*_*j*_. Thus (19) is admissible, and by suppressing the *t*’s we obtain

$$\begin{aligned} \rho^{ + } (u) - \rho^{ + } (z) & = \mathop {\sup }\limits_{{\xi \in L_{u} }} \rho (\xi ) - \mathop {\sup }\limits_{{\xi \in L_{u} }} \rho (\xi ) \\ & \le \rho (\xi^{\prime}) - \rho (\xi_{0} ) \le k|\xi^{\prime} - \xi_{0} | \le k|u - z|. \\ \end{aligned}$$

If the roles of *u* and *z* are interchanged, the proof is completed. □

In spite of considerable progress so far made in the field of population dynamics some years back, there are still many open problems. In particular, the numerical exploration of (6) for *α* > 1 has received little or no attention when the domain of interaction is considered wide enough to contain the population spread. Put together all these findings, we are motivated to seek for an appropriate and efficient numerical solution of (6) in one and two-dimensional space which we consider on an infinite domain truncated at some large, but finite value of *l*. We proceed in the next section to describe these methods.

## Numerical method

We discuss briefly the spatial discretization methods used in in this paper. When a time dependent partial differential equation is discretized in space especially with either a finite difference or spectral approximations, it results to system of coupled ordinary differential equations in time, the resulting ODEs coming from the notion of method of lines (MOL) (Owolabi and Patidar 2014a) is stiff, such a system requires the use of higher-order approximation scheme in both space and time since naturally some of these time-dependent problems are found of combining lower-order nonlinear terms with higher-order linear terms. In one-dimension, we consider the semi-linear partial differential equation

20 $$\begin{array}{*{20}l} {\frac{\partial u}{\partial t} = D\frac{{\partial^{2} u}}{{\partial x^{2} }} + \tau u^{\alpha } ,} \hfill &\quad { - l \le x \le l,\;t > 0,} \hfill \\ {u(x,0) = u_{0} (x),} \hfill &\quad { - l \le x \le l,} \hfill \\ {u(0,t) = u(l,t) = 0,} \hfill &\quad {t > 0,} \hfill \\ \end{array}$$

with *D* > 0, *τ* > 0 and *α* > 0.

### Spatial discretization method

We discretize in space with step size *h* = *x/*(*N* − 1) and approximate the second-order spatial derivative by the fourth order central difference operator, we obtain a system of nonlinear ordinary differential equations

21 $$\frac{{du_{i,j} }}{dt} = D\left[ {\frac{{ - u_{i + 2,j} + 16u_{i + 1,j} - 30u_{i,j} + 16u_{i - 1,j} - u_{i - 2,j} }}{{12h^{2} }}} \right] + \tau (u_{i,j} )^{\alpha } ,$$

and *u* = [*u*_1_, *u*_2_,…, *u*_*l*_]*T*, for 1 ≤ *i*, *j* ≤ *l.*

 Again, the two-dimensional form of system (6) can be written as

22 $$\begin{array}{l} {\frac{\partial u}{\partial t} = D\left( {\frac{{\partial^{2} u}}{{\partial x^{2} }} + \frac{{\partial^{2} u}}{{\partial y^{2} }}} \right) + \tau u^{\alpha} ,(x,y) \in \varOmega = (l_{1} \le x, y \le l_{2} ),} \quad {t > 0,} \hfill \\ {u(x,y,0) = u_{0} (x,y),} \quad {l_{1} \le x,y \le l_{2} ,} \hfill \\ {u(0,t) = u(l_{2} ,t) = 0,} \quad {t > 0,} \hfill \\ \end{array}$$

now, we discretize the spatial domain by mesh (*x*_*i*_, *y*_*j*_) = (*l*_1_ + *i* × *h*_*x*_, *l*_1_ + *j* × *h*_*y*_) where *h*_*x*_ = (*l*_2_ − *l*_1_)*/*(*N*_*x*_ + 1), *h*_*y*_ = (*l*_2_ − *l*_1_)*/*(*N*_*y*_ + 1) and 0 ≤ *i* ≤ *N*_*x*_ + 1 and 0 ≤ *j* ≤ *N*_*y*_ + 1. Using fourth order central difference discretization on the linear term, we obtain a system of nonlinear ODEs of the form

23 $$\begin{aligned} \frac{{du_{i,j} }}{dt} & = \frac{D}{12}\left[ {\frac{{ - u_{i + 2,j} + 16u_{i + 1,j} - 30u_{i,j} + 16u_{i - 1,j} - u_{i - 2,j} }}{{h_{x}^{2} }}} \right] \\ & \quad + \frac{D}{12}\left[ {\frac{{ - u_{i,j + 2} + 16u_{i,j + 1} - 30u_{i,j} + 16u_{i,j - 1} - u_{i,j - 2} }}{{h_{y}^{2} }}} \right] + \tau (u_{i,j} )^{\alpha } \\ \end{aligned}$$

where

24 $$u = \left( {\begin{array}{*{20}c} {u_{1,1} } & {u_{1,2} } & \cdots & {u_{{1,N_{y} }} \,\,\,\,\,u_{{1,N_{y} + 1}} } \\ {u_{2,1} } & {u_{2,2} } & \cdots & {u_{{2,N_{y} }} \,\,\,\,\,\,\,\,u_{{2,N_{y} + 1}} } \\ \vdots & \vdots & \vdots & \vdots \\ {u_{{N_{x} ,1}} } & {u_{{N_{x} ,2}} } & \cdots & {u_{{N_{x} ,N_{y} }} \,\,\,\,\,\,\,\,u_{{N_{x} ,N_{y} + 1}} } \\ \end{array} } \right)_{{N_{n} \times N_{y} + 1}}$$

Spatial discretisation of Eq. (6) can also be done using Fourier spectral method with periodic boundary conditions (Boyd 2001; Craster and Sassi 2006; de la Hoz and Vadilo 2008; Kassam and Trefethen 2005; Trefethen 2000; Weideman and Reddy 2001). We adapt the Fourier spectral method from (Trefethen 2000) and applied it to (6), leaving all the time stepping in Fourier space gives the following system of ordinary differential equations

25 $$\hat{u}_{t} = - Dk^{2} \hat{u} + \tau (\hat{u})^{\alpha } ,$$

so that the linear term of (6) now becomes a diagonal. Next, the systems (21), (23) and (25) will now be integrated using a time integration method as explained below.

At this junction, we have discretised the system (6) in spatial variables with both finite difference approximations and spectral approximations, we can now present the system of

ODEs obtained in the form

26 $$\begin{aligned} & u_{t} = Lu + F(u,t),\quad t > 0, \\ & u(x,0) = u_{0} . \\ \end{aligned}$$

Let us use the idea of the so-called abstract ODEs in Hilbert space (*H*) to nonlinear problem (26). Let us take an assumption that the linear operator *L*:*D*(*L*) *⊂* *H* *→* *H* is defined by

$$Lu = \sum\limits_{j = 1}^{\infty } {\lambda_{i} \langle u,\psi_{j} \rangle \psi_{j} ,\quad u \in D(L)}$$

where *ψ*_*j*_, *j* = 1, 2,*…* is the complete orthonormal system in (*H*), it follows that

$$0 > \lambda_{1} > \lambda_{2} > \cdots ,\quad\mathop {\lim }\limits_{j \to \infty } \lambda_{j} = - \infty .$$

Following this assumption, we require powers (−L)^*δ*^ for 0 ≤ *δ* ≤ 1. In that

$$H_{\delta } = D(( - L)\delta )\quad {\text{and}}\quad \| u\|_{\delta } = \| ( - L)\delta )u\| .$$

Known, (−*L*)^*δ*^ as (*L*) is regarded as closed operator, and (*H*_*δ*_) is a Banach space w.r.t graph norm ∥*u*∥ + ∥*u*∥_*δ*_. We have

$$\begin{aligned} ||u||^{2} & = \sum\limits_{j = 1}^{\infty } {\langle u,\psi_{j} \rangle^{2} } \\ & \quad \le \left( { - \lambda_{1} } \right)^{ - 2\delta } \sum\limits_{j = 1}^{\infty } {\left( { - \lambda_{j} } \right)}^{2\delta } \langle u,\psi_{j} \rangle^{2} \\ & \quad = \left( { - \lambda_{1} } \right)^{ - 2\delta } ||u||_{\delta }^{2} . \\ \end{aligned}$$

Due to Parseval’s equation, we have from the first inequality due to 0 > *λ*1 > *λ*2 >···, lim_*j→∞*_*λ*_*j*_ = −*∞* that (−*λ*_*j*_)^2*δ*^*/*(−*λ*_1_)^2*δ*^ ≥ 1, which means that the graph norm ≡ to the norm ∥*u*∥_*δ*_ which can be used as a norm on *H*_*δ*_.

#### **Lemma 4**

*Let* 0 ≤ *δ* ≤ 1, *it follows that*

$$\begin{array}{l} {\left\| {\left( { - L} \right)^{\delta } e^{Lt} } \right\| \le c_{\delta } t^{ - \delta } ,} \quad {t > 0,} \hfill \\ {\left\| {e^{Lt} u - u} \right\| \le t^{\delta } \left\| {\left( { - L} \right)^{\delta } u|} \right\|,} \quad {t \ge 0,\;u \in H_{\delta }.} \\ \end{array}$$

#### *Proof*

For all *x* *∈* R, we choose *ex* ≤ *e*^*x*^*, x* = −*λ*_*j*_*t/δ*, we obtain

$$\begin{aligned} - e\lambda_{j} t\delta & \le e^{{ - \frac{{\lambda_{j} t}}{\delta }}} \\ & \Leftrightarrow - e^{{\frac{{\lambda_{j} t}}{\delta }}} \frac{{\lambda_{j} t}}{\lambda }e \le 1 \\ & \Leftrightarrow - \lambda_{j} e^{{\frac{{\lambda_{j} t}}{\delta }}} \le \left( {\frac{\lambda }{e}} \right)\frac{1}{t} \\ & \Leftrightarrow \left( { - \lambda_{j} } \right)^{\delta } e^{{\frac{{\lambda_{j} t}}{\delta }}} \le \left( {\frac{\lambda }{e}} \right)t^{ - \delta } . \\ \end{aligned}$$

From the definition,

$$( - Lu)^{\delta } e^{Lt} u = \sum\limits_{j = 1}^{\infty } {(\lambda_{j} )^{\delta } e^{{\lambda_{j} t}} \langle u,\psi_{j} \rangle \psi_{j} } ,$$

and by using the Parseval equation, we have

$$\begin{aligned} ||( - Lu)^{\delta } e^{Lt} u||^{2} & = \sum\limits_{j = 1}^{\infty } {|(\lambda_{j} )^{\delta } e^{{\lambda_{j} t}} \langle u,\psi_{j} \rangle \psi_{j} |} \\ & \le \underbrace {{(\lambda_{j} )^{\delta } e^{{\lambda_{j} t}} }}_{{ \le s_{1} }}\underbrace {{\sum\limits_{j = 1}^{\infty } {|\langle u,\psi_{j} \rangle \psi_{j} |} }}_{{s_{2} }} \\ \end{aligned}$$

where *s*_1_ = (*δ/e*)^*δ*^ *t*^−*δ*^ and *s*_2_ = ∥*u*∥^2^. Taken together, we have

$$\left\| {( - L)^{\delta } e^{Lt} } \right\|\; \le \;c_{\delta } t - \delta .$$

### Time-stepping method

By adopting the description in (Cox and Matthews 2002; Du and Zhu 2005; Kassam and Trefethen 2005; Krogstad 2005; Owolabi and Patidar 2016), Cox and Matthews fourth order exponential time differencing Runge–Kutta formula (Cox and Matthews 2002) was used to advance the resulting ODEs are:

27 $$\begin{aligned} u_{n + 1} & = u_{n} e^{Lh} + F(u_{n} ,t_{n} )\left[ { - 4 - Lh + e^{L\Delta t} \left( {4 - 3Lh + L^{2} h^{2} } \right)} \right] \\ & \quad + 2(F(a_{n} ,t_{n} + h/2) + F(b_{n} ,t_{n} + h/2))\left[ {2 + Lh + e^{Lh} ( - 2 + Lh)} \right] \\ & \quad + F(c_{n} ,t_{n} + h)\left[ { - 4 - 3Lh - L^{2} h^{2} + e^{Lh} (4 - Lh)} \right]/L^{3} h^{2} , \\ \end{aligned}$$

where

$$\begin{aligned} a_{n} & = u_{n} e^{Lh/2} + \left( {e^{Lh/2} - I} \right)F(u_{n} ,t_{n} )/L, \\ b_{n} & = u_{n} e^{Lh/2} + \left( {e^{Lh/2} - I} \right)F(a_{n} ,t_{n} + h/2)/L, \\ c_{n} & = u_{n} e^{Lh/2} + \left( {e^{Lh/2} - I} \right)(2F(b_{n} ,t_{n} + h/2) - F(u_{n} ,t_{n} ))/L, \\ \end{aligned}$$

where for instance, if (27) is used in conjunction with ODE (25), *L* is the linear diffusion term, −*Dk*^2^ and *F* represents the term *τ uα* which could be linear or nonlinear.

### Stability analysis

**Fig. 1:**
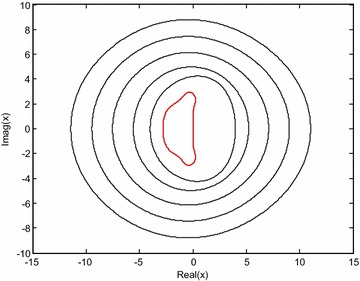
The boundary of stability regions of the ETDRK4 method in the complex plane *x* for some negative values of *y*

We follow the general stability idea as discussed in (Beylkin et al. 1998; Cox and Matthews 2002; Fornberg and Driscoll 1999; Owolabi and Patidar 2014b) for the numerical scheme that incorporates the use of different methods for the treatment of both the linear and nonlinear parts of Eq. (26). To examine the stability of ETDRK4 method (27), we linearize the nonlinear autonomous ODE

28 $$\dot{u} = gu\left( t \right) + F(u,t),$$

about a fixed point *u*_0_, such that *gu*_0_ + *F* (*u*_0_) = 0. As a result of linearizing, we obtain

29 $$\dot{u} = gu\left( t \right) + \lambda \left( {u,t} \right),$$

where *u* is the perturbation to *u*_0_ and *λ* = *F′*(*u*, *t*) at *u*(*t*) = *u*_0_ is either a diagonal or block matrix that contains the eigenvalues of *F*. For the fixed point *u*_0_ to be stable, it is required that *Re*(*g* + *λ*) < 0. On applying the ETDRK4 method to (29), a recurrence relation involving *u*_*n*_ and *u*_*n*_ + 1 is obtained (de la Hoz and Vadilo 2008). By introducing the notation *x* = *λh*, y = *gh*, the amplification factor is given as

30 $$\frac{{u_{n + 1} }}{{u_{n} }} = r(x,y) = g_{0} + g_{1} x + g_{2} x^{2} + g_{3} x^{3} + g_{4} x^{4}$$

where

31 $$\begin{aligned} g_{1} & = 1 + y + \frac{y}{2} + \frac{{y^{3} }}{6} + O(y^{4} ), \\ g_{2} & = \frac{1}{2} + \frac{y}{2} + \frac{{y^{2} }}{4} + \frac{{247y^{3} }}{2880} + O(y^{4} ), \\ g_{3} & = \frac{1}{6} + \frac{y}{6} + \frac{{61y^{2} }}{720} + \frac{{y^{3} }}{36} + O(y^{4} ), \\ g_{4} & = \frac{1}{24} + \frac{y}{32} + \frac{{7y^{2} }}{640} + \frac{{19y^{3} }}{11520} + O(y^{4} ). \\ \end{aligned}$$

obviously, as *y* *→* 0, approximation in (31) reduces to

32 $$r(x) = 1 + x + \frac{{x^{2} }}{2} + \frac{{x^{6} }}{6} + \frac{{x^{4} }}{24} ,$$

which corresponds to the amplification factor of fourth-order Runge–Kutta method. Hence, we continue with our analysis by taking the real negative values of *y* in the complex *x* plane where *|r|* < 1, by setting *r* = *e*^*iθ*^, with *θ ∈* [0, 2*π*]. The curves in Fig. 1 correspond to *y* = 0, −3.5, −5, −7, −9, −11 from the inner curve to the outer curve. It is noticeable that the stability region of the inner curve obtained at *y* = 0, coincides with the stability region of classical fourth-order Runge–Kutta method.

## Numerical simulations

To examine the efficiency and accuracy of our approach for ETDRK4 methods, we consider the numerical simulations of system (6) in one and two dimensions. We further justify the supremacy of ETDRK4 in comparison with the existing standard schemes of higher-orders by reporting the root mean square norm error of the solution defined by

33 $$||L_{2} || = \sqrt {\frac{{\sum\nolimits_{j = 1}^{n} {(e_{j} - c_{j} )^{2} } }}{{\sum\nolimits_{j = 1}^{n} {(e_{j} )^{2} } }}} ,$$

respectively, where *e*_*j*_ and *c*_*j*_ are the exact and computed values of the solution *u* at point *j*, and *n* is the number interior points.

### Test 1: one-dimensional nonlinear KiSS model

In one-dimension, we consider the KiSS model of Kierstead and Slobodkin (1953) and Skellam (1951), subject to initial and zero-flux boundary conditions

34 $$\begin{array}{*{20}l} {\frac{\partial u}{\partial t} = D\frac{{\partial^{2} u}}{{\partial x^{2} }} + \tau u^{\alpha } ,} \hfill & { - l \le x \le l,\;t > 0,} \hfill \\ {u(x,0) = \sin (2\pi x),} \hfill & { - l \le x \le l,} \hfill \\ {u(0,t) = u(l,t) = 0,} \hfill &\quad {t > 0,} \hfill \\ \end{array}$$

where *u*(*x*, *t*) is the density of the organisms at spatial domain *x* and time *t*, *τ* and *α* are both positive parameters, and *D* is the diffusion coefficient that measures the rate of dispersal. The particular choice of boundary conditions indicates that the organisms cannot boom or live beyond the domain. This assumption is taken to ensure that the experiment is not influenced by any external factor. All simulations here run for *N* = 200.

**Fig. 2:**
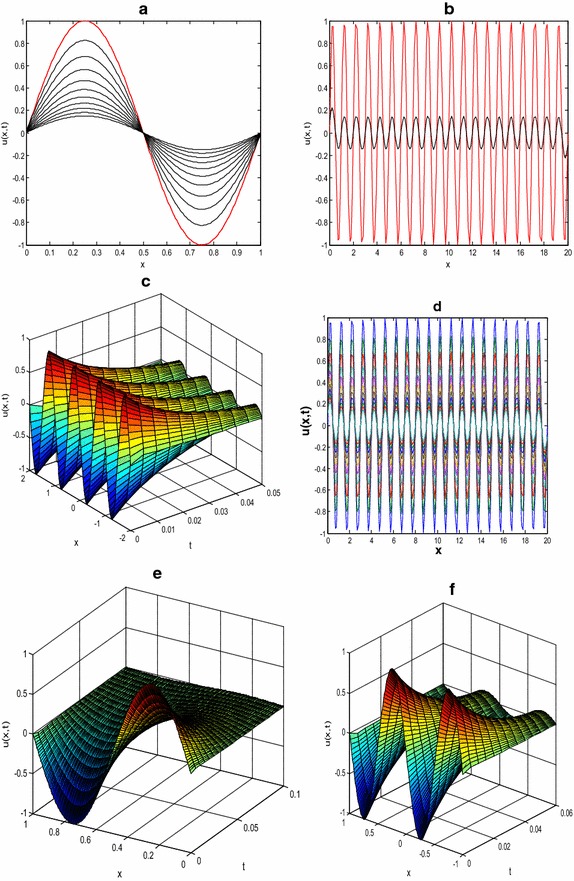
Numerical solutions of one-dimensional KiSS model (34). Time dependent density profiles *u*(*x*, *t*) versus position *x* on interval −*l* ≤ *x* ≤ *l* for *D* = *τ* = *α* = 1

In Fig. 2, the successive profile in (a) is obtained at *T* = 0.01(0.005)0.05. Panel (b) and the contour plots (d) are obtained for [0, 20], *T* = 0.05. Surface plot (c) is obtained at parameter values *T* = 0.05, in the interval [−2, 2]. Panels (e) is obtained at parameter value *T* = 0.1 in the interval [0, 1] while (f) obtained at final time *T* = 0.05 on domain [−1, 1]. The results presented have shown various behavioral patterns that could evolved when the patch size of one-dimensional KISS model (34) is varied in spatial domain.

**Fig. 3:**
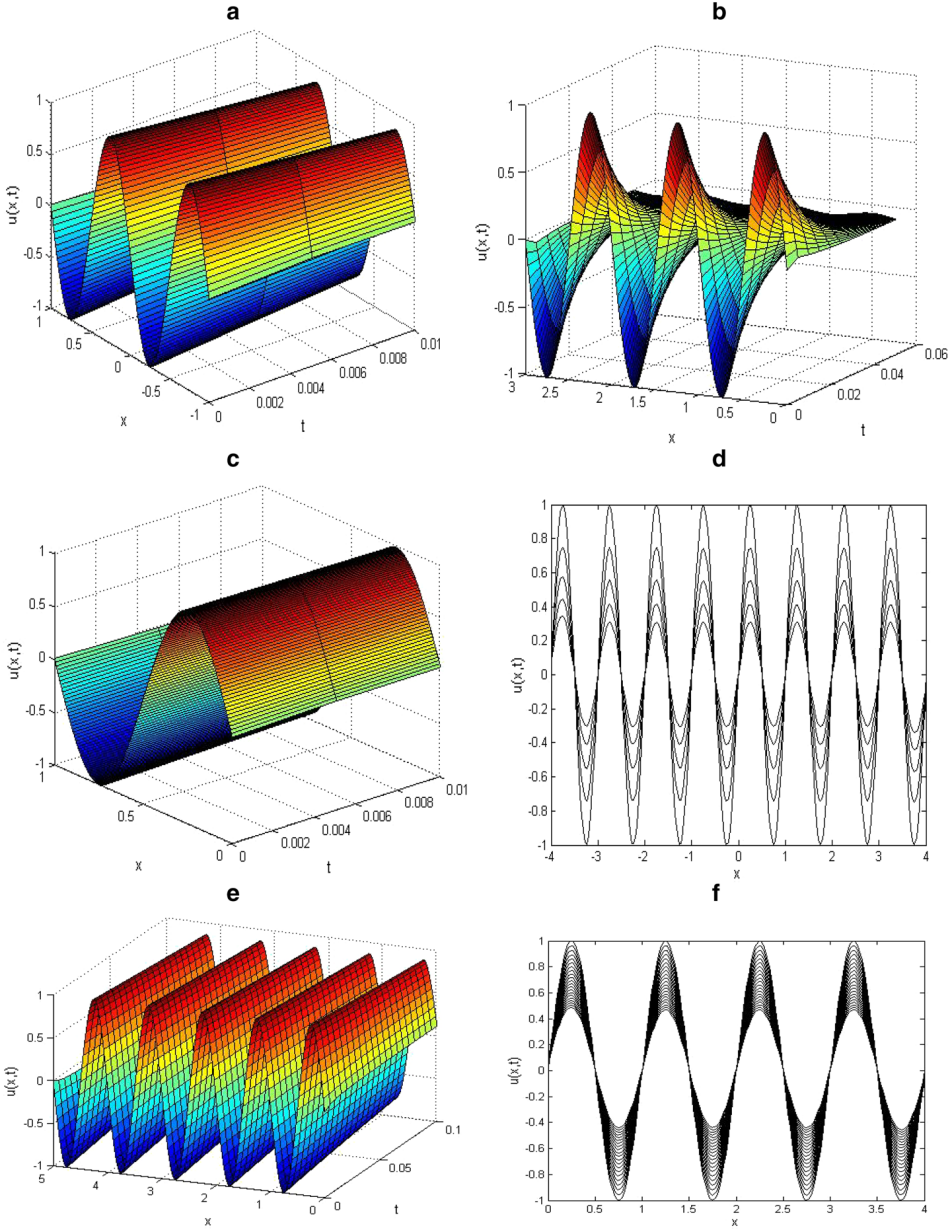
Diffusion-driven spatial patterning in one-dimensional KISS model (34) as it changes with both spatial domain *x* with varying time *t*

The plots in Fig. 3 indicate the results from initial time (*t*0) to final time *T* showing the density profiles *u*(*x*, *t*) versus position *x* on a closed interval −*l* ≤ *x* ≤ *l* for the choice of growth rate *τ* = 0.5 and critical exponent *α* = 2. The successive profile in (a) is obtained at *T* = 0.01 with *D* = 0.5 on [−1, 1]. For panel (b), large *D* = 2, *T* = 0.05 on [0, 3]. For (c), *D* = 0.1, *T* = 0.01 on [0, 1]; plots (d) is obtained on [−4, 4], *T* = 0.02 for large diffusion coefficient *D* = 1.5. Panel (e) is obtained on the spatial domain [0, 5] with *T* = 0.1 and *D* = 0.05. Contour plot (f) is obtained with parameter values *D* = 0.2, *T* = 0.1 on domain of size [0, 4]. The results presented here have equally revealed some of the dispersal-driven patterns that arise as a result of diffusion.

**Fig. 4:**
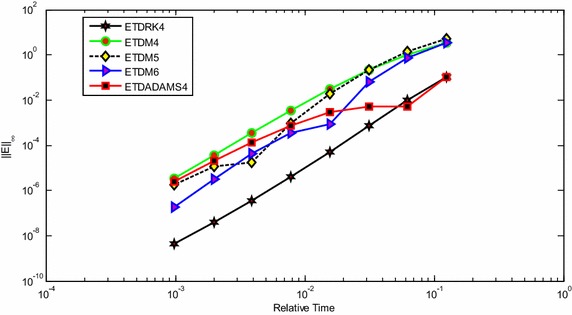
Performance of ETDRK4, ETDM4, ETDM5, ETDM6 and ETDADAMS4 methods for solving the KISS Eq. (34) with parameter values *T* = 1, *D* = 0.5, *τ* = 0.5 and *α* = 2 on interval [−1, 1] for *N* = 200

It is clear from the result presented in Fig. 4, that ETDRk4 has the best convergence when compared to other exponential time differencing schemes, such as ETDM4, ETDM5, ETDM6 and ETDADAMS4 methods (for details of these schemes, see Hochbruck and Ostermann 2011; Hochbruck et al. 2010). In Table 1, we illustrate the tradeoff between the computational [CPU (s)] time and the accuracy as time step *k* is refined for each of the methods with parameter values *T* = 1, *D* = 0.5, *τ* = 0.5 and *α* = 2 on interval [−1, 1] for *N* = 200. We display accuracy as a function of CPU time respectively for each of the schemes, to add a competing factor in differentiating between the methods.

**Table 1:** Error norm ||*L*_2_|| at some selected time steps for solving KiSS Eq. (34)

Method	Time step (k)	||*L*_2_||	CPU time (s)
ETDM4	1/32	0.2150	1.5086
	1/64	0.0301	1.5467
	1/256	3.3910e−004	1.7189
	1/1024	3.4784e−006	3.8817
ETDM5	1/32	0.2170	1.9896
	1/64	0.0195	2.0277
	1/256	1.7793e−005	2.3538
	1/1024	1.7923e−006	4.6010
ETDM6	1/32	0.0664	2.0405
	1/64	8.5299e−004	2.0515
	1/256	4.2840e−004	2.2500
	1/1024	1.8248e−007	8.2387
ETDADAMS4	1/32	0.0050	1.4850
	1/64	0.0028	1.5511
	1/256	1.3334e−004	1.8339
	1/1024	2.3963e−006	4.4020
ETDRK4	1/32	7.2013e−004	1.1146
	1/64	5.0921e−005	1.0236
	1/256	3.5038e−007	1.3198
	1/1024	4.4086e−009	3.8726

### Test 2: two-dimensional nonlinear KiSS model

Our major aim in this paper is to examine the behavior of system (6) numerically in two-dimensional space, that is, when the Laplacian operator *∇*^2^ = *∂*^2^*/∂x*^2^ + *∂*^2^*/∂y*^2^. One-dimensional form of KiSS equations are relatively simple to undertake using method of lines coupled with spatial adaptive schemes. In-fact, solutions of the form (6) have been sought theoretically (Allen 2007; Kot 2001; Okubo 1978). Unfortunately, in two space dimensions, numerical solutions of KiSS model (22) still requires some attentions, since simulations based upon the more conventional ideas become more time consuming. In the spirit of (Owolabi 2015a; Owolabi and Patidar 2015, 2016), we consider the two-dimensional case

35 $$\begin{array}{l} {\frac{\partial u}{\partial t} = D\left( {\frac{{\partial^{2} u}}{{\partial x^{2} }} + \frac{{\partial^{2} u}}{{\partial y^{2} }}} \right) + \tau u^{\alpha } ,\quad } {(x,y) \in \varOmega = (l_{1} \le x,y \le l_{2} ),\;t > 0,} \hfill \\ {u(x,y,0) = u_{0} (x,y),}\quad {l_{1} \le x,y \le l_{2} ,} \hfill \\ {u(0,t) = u(l_{2} ,t) = 0,} \quad {t > 0,} \hfill \\ \end{array}$$

where *u*(*x*, *y*, *t*) is the density of organisms at spatial coordinates *x*, *y* and time *t*. *D* > 0 remains the diffusion coefficient, while *τ* > 0 and *α* ≥ 1 are the respective growth rate and critical exponent.

**Fig. 5:**
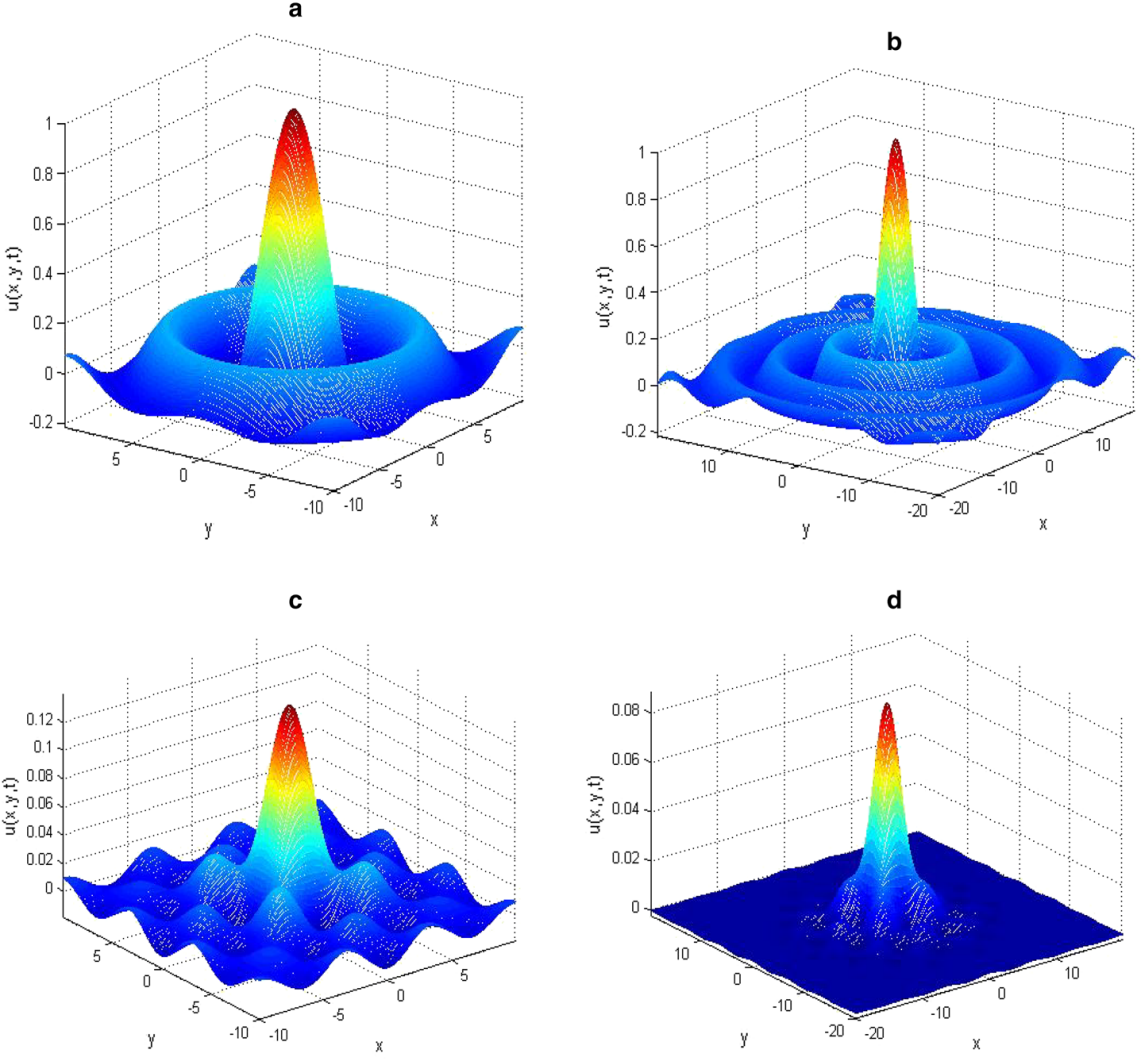
Solutions of two-dimensional KISS model (35) on different spatial domain. The initial and parameter values: *u*0(*x*, *y*) = sinc[√(*x/π*)^2^ +√ (*y/π*)^2^], with *D* = 1, *τ* = 0.01, *T* = 0.5 and *α* = 2 on **a** *l* = 10 and **b** *l* = 20. Plots **c** *T* = 1.2, *l* = 10 and **d** *T* = 2, *l* = 20 are obtain with initial data *u*0(*x*, *y*) = cos(*x*) cos(*y*) exp(−√(*x* ^*2*^2 + *y* ^*2*^ */*4))

**Fig. 6:**
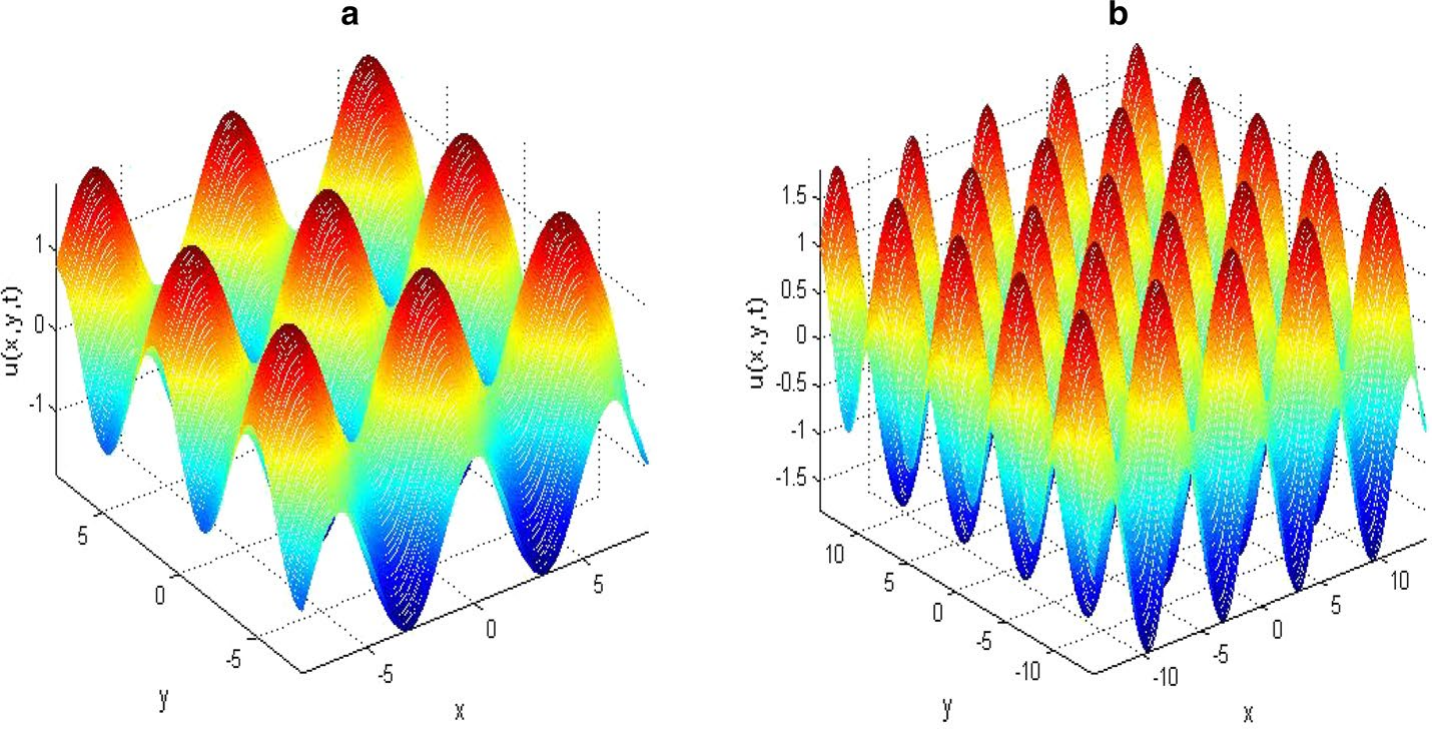
Solutions of two-dimensional KISS model (35) on different spatial domain. The initial and parameter values: *u*0(*x*, *y*) = exp(1*/*10)(cos *x* + sin *y*), with *T* = 0.05 *D* = 1, *τ* = 0.01 and *α* = 2 on **a** *l* = 8 and **b** *l* = 14

**Fig. 7:**
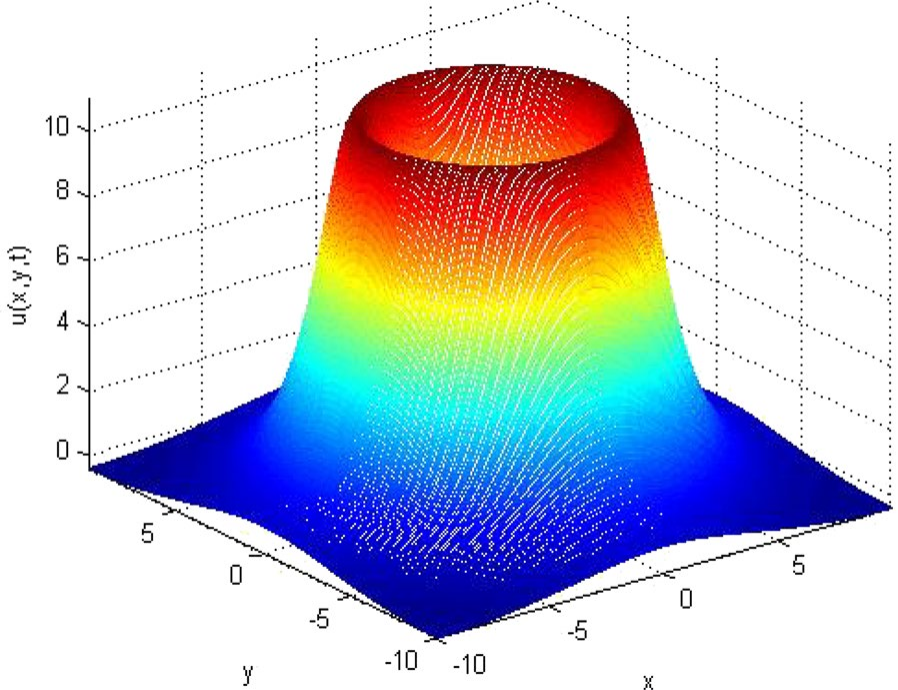
Solutions of two-dimensional KISS model (35) on different spatial domain. The initial and parameter values are: *u*_0_ = sin(√(*x/π*)^2^ +√(*y/π*)^2^), with *D* = 1, *τ* = 0.01, *α* = 2 and *T* = 1 for *l* = 10

The initial data and parameter values were carefully chosen to make the Figures replicate some of the existing patterns. In all cases, the space step *h* was kept equal to *l*, that is, *h*_*x*_ = *h*_*y*_ = *l* in the spatial domain −*l* ≤ *x*, *y* ≤ *l*. Figures 5, 6, 7 show various patterns formation process that could emerge as a result of variation of the initial data.

The two-dimensional results presented in Figs. 5, 6, 7 are much more meaningful in the contexts of mathematical biology and ecology. It is clear from the numerical results that the increase in the spatial domain *l* is the factor responsible for the spread of the phytoplankton plants living in the ocean.

## Conclusion

In this paper, we have further justified the assertion made by Kassam and Trefethen (2005) on the efficiency and suitability of ETDRK4 schemes in conjunction with spatial discretization methods by comparing it with exponential time differencing method (ETDADAMS4) of Adams type and exponential time differencing multistep (ETDM4, ETDM5, ETDM6) methods all are of the higher orders. This approach was tested with the reaction–diffusion equation, a nonlinear form of KiSS model that was named after Kierstead and Slobodkin (1953) and Skellam (1951), which was originally developed to investigate the size of nutrient patches needed to sustain phytoplankton blooms. We carried out numerical simulations in both one- and two-dimensional space on spatial domain *x* *∈* [−*l*, *l*], that are chosen large enough to support the boom. Our numerical results revealed that the population size increases if the domain size *l* also increased. Some initial data and parameter values are chosen to mimic some existing patterns. The methodology presented in this paper can be extended to higher-order fractional derivative (Atangana and Nieto 2015; Atangana 2015) and time-dependent parabolic problems.
